# The role of short-term memory, type of practice and metacognitive judgments in predicting cognitive offloading

**DOI:** 10.3389/fcogn.2025.1595956

**Published:** 2025-07-04

**Authors:** Dan Chiappe, Kim-Phuong L. Vu, Michelle Tornquist

**Affiliations:** ^1^Department of Psychology, California State University, Long Beach, CA, United States; ^2^Department of Psychology, University of Liverpool, Liverpool, United Kingdom

**Keywords:** offloading, short-term memory, metacognition, practice effects, perseveration

## Abstract

We examined whether short-term memory (STM) capacity, type of practice, metacognitive judgments and task characteristics influence the likelihood of cognitive offloading. We used a Letter-Naming task, where people hear sets of letters they subsequently must report. We manipulated set size (i.e., 2, 4, 6, 8, and 10 letters) and whether people could write down the letters as they heard them prior to reporting them. We also manipulated the difficulty of the practice trials by varying their set sizes. Consistent with previous studies, we found participants offloaded more as set size increased and that offloading increased accuracy, especially for the higher set sizes. Difficult practice also increased offloading, particularly for smaller set sizes, with many participants developing a perseveration strategy in favor of offloading. Moreover, STM capacity was negatively correlated with frequency of offloading in the intermediate but not in the smallest or largest set sizes. Metacognitive judgments and self-ratings of effort and motivation revealed that although motivation to correctly report the letters predicted overall frequency of offloading, judgments of effort involved in offloading and confidence in task performance did not. Finally, removing the ability to offload also led to lower estimates of short-term memory ability and decreased motivation to correctly report letters.

## 1 Introduction

Cognitive offloading is using physical actions in the world to limit costly internal storage and processing (Gilbert, 2024; Risko and Gilbert, 2016). Examples from daily life include putting a note by the door to remember to bring something, writing down a phone number, or entering an appointment reminder in a calendar. The decision to offload reflects task demands and the availability of cognitive resources (e.g., Chiappe et al., 2016; Gilbert, 2024; Meyerhoff et al., 2021; Morrison and Richmond, 2020). In the current study, we examined whether short-term memory (STM) capacity determines the likelihood people will offload information. We also examined whether practice conditions that encourage greater offloading lead to a greater frequency of offloading during experimental trials. In addition, we tested whether participants' self-ratings of effort and motivation and metacognitive judgments of confidence predict offloading frequency. Finally, we examined how the ability to offload influences perceptions of task performance, task motivation, and self-perceptions of short-term memory ability.

### 1.1 Offloading as a strategic process

Prior research on cognitive offloading has focused primarily on the decision-making processes and task characteristics that influence offloading, as well as the consequences of doing so (Gilbert et al., 2023; Risko and Gilbert, 2016). For example, in the case of memory, Storm and Stone (2015) found that being able to offload a list of words by saving them on a computer decreased proactive interference for learning a new set of words. Furthermore, in a prospective memory task manipulating whether people could offload intentions, Gilbert (2015) found setting external reminders improved performance, and that people were more likely to offload as the task became more difficult. Fellers et al. (2023) also showed that being instructed to set external reminders increased the likelihood of participants completing future tasks on time compared to those not told to set a reminder.

Many studies also reveal that the decision to offload is strategic. It considers multiple factors, including the cost associated with offloading information. Grinschgl et al. (2021) demonstrated this using the Pattern Copy Task (PCT), where participants had to copy a colored pattern of objects shown on the bottom-left of the screen onto an empty workspace window located on the top-right of the screen by dragging and dropping objects contained in an array onto the workspace window. The window containing the model had to be opened to see the pattern. Offloading was assessed by the number of openings of the model window, with fewer openings indicating that participants are storing more information internally. They varied the temporal costs of offloading by manipulating whether a delay was added every time the model window was opened. They found that increasing the temporal costs lead to a decrease in offloading behavior, yielding worse performance on the PCT. Using a similar task, Patrick et al. (2015) found that type of training affected likelihood of offloading, as participants who experienced a lag in opening the window during training continued to offload less during transfer trials, even though those did not feature a delay.

In line with the strategic nature of offloading, Weis and Wiese (2019) have shown that this behavior also varies with task goals. They used an extended rotation task where participants had to state whether two patterns that differ in angle of rotation are the same or different. They were given the option to offload the mental rotation process by using a physical knob that allowed them to rotate one of the stimuli on the screen. They found that when participants were told to prioritize speed, they offloaded less than when they were told to prioritize accuracy. Indeed, this shift in offloading led to high goal-related performance, i.e., fast answers in the speed goal condition and more accurate answers in the accuracy goal condition.

Gilbert (2024) has offered a mechanistic model of cognitive offloading. He argues that it involves a value-based decision-making process that works on two principles: first, storing an item in internal memory comes with an opportunity cost, due to its limited capacity. Storing one piece of information interferes with the ability to store other information. Second, although it avoids the opportunity cost, offloading generally involves a small cost, due to the physical effort and time involved in creating external reminders. When deciding whether to offload, a person weighs the value of remembering the information (which can be more or less important) against both costs, opting for the strategy that yields the greatest benefit. This model can explain, for example, why participants prefer to offload high value information when given the opportunity to offload (e.g., Dupont et al., 2023), as the benefits far outweigh the costs of offloading compared to the opportunity costs of storing the information internally. It can also explain why increasing the costs of offloading decreases the likelihood of doing so (e.g., Grinschgl et al., 2021), as the net value associated with offloading decreases.

### 1.2 Prior studies on WM/STM and offloading

Research examining whether individual differences influence offloading behavior has mainly focused on the role of working memory (WM) and short-term memory (STM) capacity. Most memory models hold that STM is a component of WM (e.g., Baddeley, 2000; Cowan, 2008), a difference reflected in the tasks used to measure the two. STM tasks require the retention of information for short periods of time, while WM tasks require not just the retention of goal-relevant information but also involve an attention-demanding processing component. WM and its storage buffers are limited in capacity. Their influence on offloading can therefore be accommodated within Gilbert's (2024) value-based decision-making model. This is because, everything else being equal, the more limited their capacity, the greater the opportunity costs associated with storing information internally, leading to a greater payoff for offloading information.

Spearheading the study of the role of STM capacity in cognitive offloading, Risko and Dunn (2015) conducted an experiment where participants completed a Letter-Naming task. This is a STM task and not a WM task because it lacks simultaneous processing requirements. The task consisted of the auditory presentation of a set of letters, sets varying in size (set sizes: 2, 4, 6, 8, and 10). Participants had to type the letters into the computer at the end of each set. Prior to each trial they were shown the size of the upcoming set. In the Choice condition, which always appeared first, participants were given the opportunity to write down the letters as they heard them, and they were allowed to refer to it when entering the letters into the computer. In the No Choice condition, participants had to rely only on internal memory. STM capacity was assessed by mean performance in the No Choice condition. They found that as set size increased, the likelihood of offloading increased. Moreover, they found that STM capacity was negatively correlated with the overall likelihood of offloading the letters.

In a second experiment examining metacognitive judgments, Risko and Dunn (2015) found that although people predicted that when relying on internal memory alone their recall accuracy would decrease with set size, they nonetheless overestimated their ability to remember, particularly for longer strings of letters. They also judged that offloading benefits accuracy, stating that they would be more likely to offload the letters as set size increases. Finally, participants judged that effort to offload the letters would be lower than the effort required to store the letters internally, a finding consistent with Gilbert's (2024) model.

Although other studies have found relations between WM and frequency of offloading (e.g., Meyerhoff et al., 2021 using an intention offloading task and a pattern copy task), not everyone has found that WM and STM predict offloading. Notably, Morrison and Richmond (2020) sought to replicate Risko and Dunn (2015) using the Letter-Naming task, but with a much larger sample size. Moreover, although STM capacity was assessed by performance on the No Choice task, WM was assessed with an OSPAN task and a Symmetry Span task. They found that frequency of offloading in the Choice condition increased with set size, but STM did not predict overall frequency of offloading. The WM tasks, however, did not account for additional variance in offloading behavior. The authors conclude cognitive offloading is not just beneficial for those with more limited WM but is of use for those with a wide range of cognitive abilities.

More recently, Richmond et al. (2025) examined the role of WM in both an intention offloading task and the Letter-Naming task. Participants performed both types of tasks under three conditions: forced internal, forced external, and free choice, presented in a fixed order. The forced internal condition required participants to use only internal memory to do the task, while the forced external condition required that participants always offload. The free choice condition left it up to participants to decide on any given trial whether to offload. The Letter-Naming task was modified to include only set sizes 2, 4, 6, and 8. For their analysis they grouped these into high (6 and 8) and low (2 and 4) memory loads. WM was assessed by combining scores for a modified version of the OSPAN task and the Symmetry Span task.

They found that relative to the forced internal condition, participants were more accurate on the tasks when either forced to offload or when given the choice to offload. With respect to WM, participants with lower WM capacity benefited more in terms of accuracy in both tasks when offloading was required, specifically for the high load condition. When given free choice to offload, those lower in WM capacity also benefited more in terms of the accuracy in the intention offloading task, regardless of memory load. In the Letter-Naming task, however, the benefit was greater for those lower in WM, but only in the high load condition. In general, then, the opportunity to offload was most beneficial for those lower in WM and this benefit increased with increasing memory load. Most importantly for our purpose, however, they did not find that WM capacity correlated with the actual *frequency* of offloading in either the high or low memory load of the Letter-Naming task or the intention-offloading task.

### 1.3 Current study

We examined how STM, type of practice, task-characteristics, metacognitive judgments and self-ratings of effort and motivation affect offloading in the Letter-Naming task. We focused on STM to examine specifically how individual differences in the limitations of the temporary storage buffer relate to the likelihood of offloading. In WM tasks, this limitation is conflated with other factors, such as variability in executive control (McCabe et al., 2010). We used the Letter-Naming task of Risko and Dunn (2015) using set sizes 2, 4, 6, 8 and 10. STM was assessed in terms of performance in the No Choice condition. Although Risko and Dunn (2015) found that STM capacity was correlated with overall frequency of offloading, Morrison and Richmond (2020) failed to replicate this. Both papers, however, looked at whether STM predicted the *overall* amount of offloading, the latter also examining whether WM predicts offloading frequency. In the present study, we carried out more fine-grained analyses, looking at whether STM was correlated with offloading not just overall, but at each of the set sizes.

Richmond et al. (2025, cited earlier) address this issue as well, but their study has some limitations from the perspective of our current goals. First, they used a Letter-Naming task that only included set sizes up to 8, while Risko and Dunn (2015) included set size 10. This makes their task easier by eliminating the most difficult condition. Second, they divided their set sizes into two groups (i.e., high and low), instead of looking at offloading patterns for each set size individually. Combined with limiting the number of set sizes, this could obscure the relation between internal memory capacity and offloading behavior. Third, they used measures of WM, not STM. Although the latter is a component of the former, WM includes other functions, which can obscure the assessment of the limitations of storage capacity and its relation to offloading.

The present study also manipulated whether participants received an Easy or a Difficult practice prior to the Choice condition, a factor not studied in prior studies using the Letter-Naming task. In the Easy condition, participants practiced with set sizes 2, 4, and 6, while in the Difficult condition, they practiced with set sizes 6, 8, and 10. Our Difficult practice condition was designed to give participants more opportunities to offload during practice trials compared to Easy practice. This was done to assess whether strategies developed during practice are carried over to experimental trials, as observed by Patrick et al. (2015). Those in the Difficult practice might come to regard an offloading strategy as superior and may therefore subsequently apply it regardless of specific task conditions. Weis and Kunde (2024) describe this as “perseveration” and claim that it can be used to avoid the costs of decision-making and strategy switching in tasks that give people the option to rely on either internal or external processes. Furthermore, since our Choice condition consisted of two blocks of trials, we were able to examine whether any differences in offloading resulting from the two practice conditions are evident throughout the task, or more pronounced early on. Indeed, Weis and Kunde (2024) found perseveration persisted across blocks of their experiments, and did not fade as the task progressed.

We also assessed metacognitive judgments to see whether frequency of offloading behavior can be predicted by people's confidence in how well they will do on the task. To this end, before the experimental trials of the Choice condition we asked participants to rate how accurate they think they will be in the upcoming trials. Many prior studies have examined how confidence in one's ability to remember influences the likelihood of offloading. Scott and Gilbert (2024), for example, found that with respect to prospective memory, participants who were less confident in their ability to remember future intentions were more likely to offload them. Moreover, Gilbert et al. (2020) found that those who were underconfident in their ability to remember future intentions were more likely to offload intentions despite receiving a maximum reward for relying on internal memory alone (see also Boldt and Gilbert, 2019 and Hu et al., 2019). However, using the Letter-Naming task, Richmond et al. (2025) failed to find judgments of confidence predicted offloading for either the high or the low memory load conditions. But their task was a shortened version that omitted the most challenging set size. Making the task easier could have attenuated any effect of confidence. We therefore tested this relationship using a more challenging version of the Letter-Naming task.

Moreover, following the Choice trials we asked participants to provide self-ratings on how much effort it had been to offload the letters and how motivated they were to correctly report the letters to see whether confidence predicts offloading frequency independent of these two factors. Effort to write the letters is likely to negatively affect the frequency of offloading according to Gilbert's (2024) model and given that participants are generally aware that offloading increases accuracy (e.g., Risko and Dunn, 2015), it is also likely that those more motivated to correctly report the letters will also be more likely to offload them.

Prior to the experimental trials of the Choice condition, we also asked participants to rate how often they will write down the letters and how much effort they think it will be to write down the letters on paper as they hear them. This allowed us to test the strategy-perseveration account of the effect of type of practice on offloading frequency. It is possible that the reason people offload more following the Difficult practice is that it has become less effortful because of greater practice at coordinating listening to the incoming letters and writing them down. Finding that following the Difficult practice participants report being more likely to offload the letters, while not judging that it is less effortful to do so, would rule out this explanation and would support the perseveration hypothesis instead.

Following the Choice and No Choice conditions we also queried the participants' judgments about how well they did and how good they rate their short-term memory. Although it is likely that participants' definitions of STM differ from those employed by cognitive scientists, these questions did allow us to assess whether the opportunity to offload information undermines people's estimates of their own cognitive abilities. Consistent with this claim, prior studies have found that when a person believes they have easy access to external information, such as through search engines, they give higher estimates of their intellectual ability (e.g., Ward, 2013; but see Kahn and Martinez, 2020). We also queried the participants regarding how motivated they were to correctly report the letters following the No Choice condition (as we did following the Choice condition). This was to determine whether making the task more difficult by removing the opportunity to offload would decrease the motivation to perform well on the task.

Given the results of Risko and Dunn (2015) and Morrison and Richmond (2020), we hypothesized the following for task characteristics influencing frequency of offloading (H1), relation between offloading and accuracy (H2), correlation of offloading with short-term memory (H3), and factors influencing metacognitive judgments and other self-ratings and their consequences for offloading (H4).

H1a: As set size increases participants will offload more information.
H1b: Participants receiving the Difficult practice will offload the letters more often during the experimental trials because of perseveration on a favored strategy developed during practice.
H2a: Accuracy will be greater overall in the Choice condition compared to the No Choice condition due to the opportunity to offload.
H2b: The differences in accuracy between the two memory conditions will be most pronounced for the largest set sizes.
In terms of the role of STM in predicting offloading in the Letter-Naming task, given the inconsistent results of Risko and Dunn (2015) and Morrison and Richmond (2020), we were agnostic regarding correlations between STM and overall offloading frequency. We did predict, however, that:
H3: STM will be negatively correlated with offloading in the intermediate set sizes. At the lowest set sizes, which most should find easy to do using internal memory, differences in STM capacity should be irrelevant. In the highest set sizes, differences in STM are also likely to be irrelevant, as most people will need to offload because the difficulty is likely to exceed the capacity of most participants. Thus, if we only assess the relationship between STM capacity and offloading behavior overall, the very easy and very difficult set sizes are likely to wash out any effects.
H4: Participants' offloading behavior will be influenced by their subjective assessment of their own abilities along with properties of the tasks. With respect to the metacognitive judgment of confidence, we predicted that low confidence in the ability to do well in the task will lead to greater offloading frequency (H4a). This would confirm prior studies that have shown that metacognitive confidence predicts overall offloading frequency in a wide range of tasks (Boldt and Gilbert, 2019; Gilbert, 2015; Scott and Gilbert, 2024). Moreover, given that prior studies (e.g., Risko and Dunn, 2015) have shown that people are aware of the benefits of offloading for performance, we also predicted that those who rated themselves more motivated to perform well will be more likely to offload during the Choice trials (H4b). We also predicted that the greater the reported effort in offloading the letters, the less likely people will be to offload them (H4c). This follows from Gilbert's (2024) decision-making model. Although several prior studies have shown that metacognitive judgments influence offloading, to our knowledge they have not examined whether confidence, effort, and motivation to respond correctly predict offloading after controlling for each other. We do so in the present study for overall offloading frequency.

Given prior studies demonstrating perseveration following Difficult practice conditions that encourage offloading strategies, we also predicted that those in the Difficult practice condition will report that they will be more likely to offload in the upcoming experimental trials relative to the Easy practice but that the two groups will not differ in their ratings of how much effort it will be to write down the letters (H4d). Finally, we predicted that following the Choice condition participants will give higher ratings to their short-term memory abilities than following the No Choice condition (H4e). We also predicted that due to the difficulty of the task, especially with the inclusion of set size 10, participants would report being more motivated to correctly report the letters in the Choice condition than in the No Choice condition (H4f).

## 2 Method

### 2.1 Participants

Students enrolled in undergraduate courses (*N* = 158) participated in exchange for course credit (*M*_age_ = 20.54, *SD*_age_ = 3.20), 138 identifying as female, 19 as male, and 1 as non-binary. Demographics of the sample were 53% Hispanic, 20% Asian, 12% White, 6% African American, 9% other or mixed ethnicity. An a priori power analysis in G^*^power indicated that the required sample size for testing the between-subjects effect of Practice Condition on offloading in an ANOVA was *N* = 128 (power criterion = 0.80, alpha = 0.05, effect size *f* = 0.25). We exceeded this, however, because as Schönbrodt and Perugini (2013) have shown, correlations only stabilize with sample sizes of 150 onwards and one of our main aims was to assess correlations between STM and offloading. After data collection was completed, we conducted a sensitivity power analysis in G^*^Power (*N* = 158; power criterion = 0.80; alpha = 0.05) for the effect of Practice Condition on offloading, which showed that our experiment was powerful enough to detect small-to-medium effects (*f* = 0.22). The present study was approved by the Institutional Review Board of California State University, Long Beach (Ref number: 23–461), which gave us consent to collect the data in this study and to publish the results. Participants provided informed consent prior to engaging in the study.

### 2.2 Materials

Following Risko and Dunn (2015), we used a Letter-Naming task administered on a Hewlett-Packard desktop computer running SuperLab 6.0 software. The task consisted of the auditory presentation of letters drawn from the following: B, C, F, H, J, K, L, M, P, Q, R, T, W, X. Set sizes ranged from 2, 4, 6, 8 and 10 letters. Before each set, the size of the forthcoming set was shown on the computer screen. Letters were then presented acoustically, one second apart. After each set, a box appeared on the screen and participants had to type in the letters that they had heard, in the order they heard them. For the Choice condition, participants were also provided with a pencil and a sheet of paper that they could use to write down the letters as they heard them, prior to typing them into the computer.

### 2.3 Procedure

When participants entered the lab, they were given the Consent Form to read and sign. Then they were presented with a demographics questionnaire. Participants were then given a set of paper and pencil personality, self-control, and emotional state questionnaires that were included for exploratory purposes and will not be reported here.^1^ After the participants completed these, they were introduced to the Letter-Naming task. Each Memory condition (Choice vs. No Choice) was made up of two blocks of 15 trials presented consecutively, for a total of 60 experimental trials. In each of the two Memory conditions, the two blocks were made up of three trials at each of the five set sizes. These were presented in random order within blocks. Prior to the experimental trials, people received either an Easy or Difficult practice set of trials. In the Easy practice, participants received nine trials made up of set sizes of 2, 4 and 6 letters, three at each level, presented in ascending order. In the Difficult practice, they received three trials at set sizes 6, 8, and 10 letters, presented in ascending order. A sheet was provided with nine lines and participants were told that they could write down the letters as they heard them if they chose to. A manipulation check revealed that those in the Easy practice condition offloaded on fewer practice trials (*M* = 2.92, *SE* = 0.32) than those in the Difficult practice condition (*M* = 8.08, *SE* = 0.20), *t*(156) = 13.83, *p* < 0.001, Cohen's *d* = 2.20.

**Figure 1:**
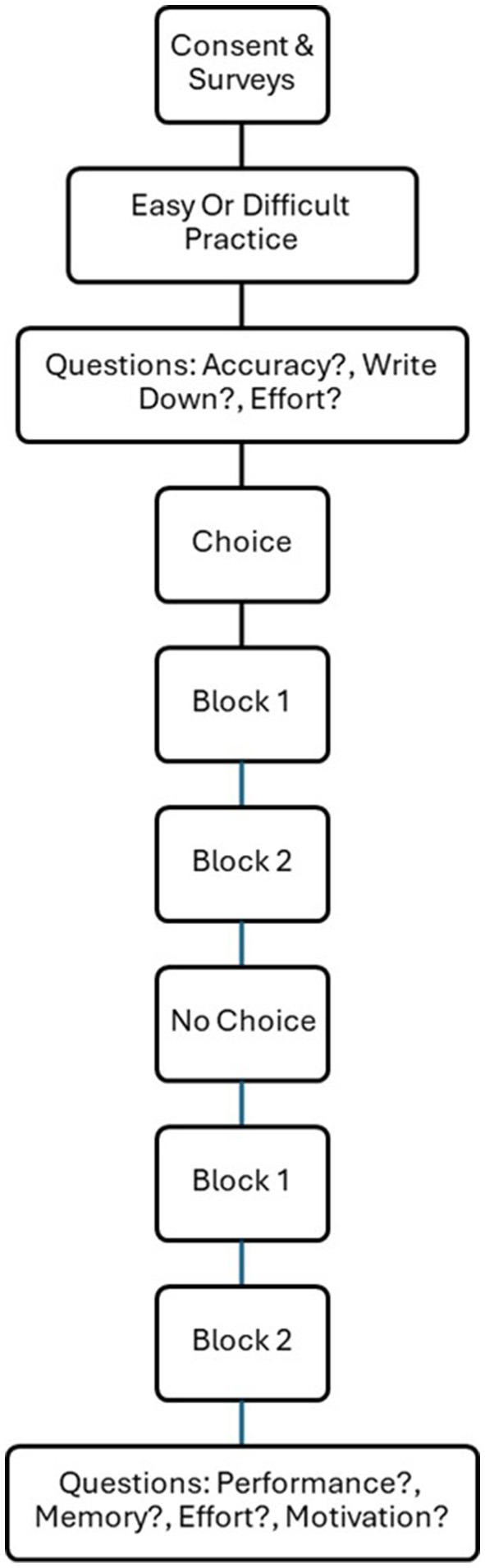
Order of tasks and conditions.

Once participants completed the practice trials the computer presented questions asking them how accurate they think they will be in the next set of trials, how often they think they will write down the letters prior to reporting them, and how much effort it will be to offload the letters. Once they answered these questions they proceeded with the 30 experimental trials. They were provided with a fresh sheet of paper that had 30 lines. The experimenters recorded on a separate sheet what the set size was and whether the participant wrote down the letters as they heard them for each trial prior to typing them into the computer. Following the Choice trials, the computer asked participants to rate their performance on the previous trials, how good they think their memory is, how much effort it was to offload the letters, and how motivated they were to correctly report the letters. After completing the Choice condition, participants were presented with the No Choice condition. Following Risko and Dunn (2015) and Richmond et al. (2025), the memory conditions were presented in a fixed order. Counterbalancing the order means that some participants would be more fatigued than others prior to the No Choice condition, affecting the assessment of STM. Given that our main goal is to examine the correlation between STM and offloading, we deemed this to be unacceptable. Moreover, since another goal was to examine the effects of practice condition on offloading, this would not be affected by failing to counterbalance the order of the memory conditions. After completing the 30 trials, the computer asked participants to rate how well they think they performed, how good their short-term memory is, and how motivated they were to correctly report the letters. Participants were then thanked for their participation and were presented with a Debrief form. Then they were escorted out of the lab. The order of tasks and conditions is summarized in Figure 1.

### 2.4 Design

The study employed a mixed design to study the effect of set size, block, and type of practice on the likelihood of offloading information, and how offloading information affects accuracy on a memory task. The repeated-measures factors were Memory Condition (Choice, No Choice), Block (Block 1, Block 2), and Set Size (2, 4, 6, 8 and 10). The between-subjects factor was Practice Condition (Easy, Difficult). The dependent variables were the number of times participants wrote down the letters as they heard them in the Choice condition and accuracy at reporting the letters at the end of each set. We also conducted confirmatory correlational analyses to examine the relation between STM and offloading at each set size and overall, and a regression analysis between metacognitive judgments of confidence, self-ratings of effort and motivation, and frequency of offloading overall on the Letter-Naming task.

## 3 Results

### 3.1 Offloading behavior

To examine whether set size, block, and type of practice affect offloading behavior, we carried out a 2 x 2 x 5 mixed ANOVA with Block (Block 1 or Block 2) and Set Size (Set Size 2, 4, 6, 8 or 10) as repeated-measures factors, and Practice Condition (Easy or Difficult) as the between-subjects factor. The DV was number of times out of 6 that they wrote down the letters prior to reporting them. Where the Mauchley test of sphericity was found to be significant, either the Greenhouse-Geisser or Huynh-Feldt correction was applied, as appropriate.

As predicted, the results revealed a main effect of Set Size, *F*(2.33, 363.98) = 295.96, *p* < 0.001, η*p*^2^ = 0.66 (Set Size 2: *M* = 0.71, *SE* = 0.09; Set Size 4: *M* = 1.10, *SE* = 0.10; Set Size 6: *M* = 2.34, *SE* = 0.08; Set Size 8: *M* = 2.82, *SE* = 0.05; Set Size 10: *M* = 2.92, *SE* = 0.03). Pairwise comparisons with Bonferroni adjustment for multiple comparisons (critical *p*-value of 0.005) revealed that each of these means was significantly different from the others with frequency of offloading increasing with set size (all *p*s < 0.001, except for Set Size 8 vs. 10, where *p* = 0.018), supporting H1a, except for the largest two set sizes. The results also revealed a main effect of Block, *F*(1,156) = 11.79, *p* < 0.001, η*p*^2^ = 0.07. Participants were more likely to write down letters in the first block (*M* = 2.02, *SE* = 0.05) than in the second block (*M* = 1.94, *SE* = 0.06) of the Choice condition.

**Figure 2:**
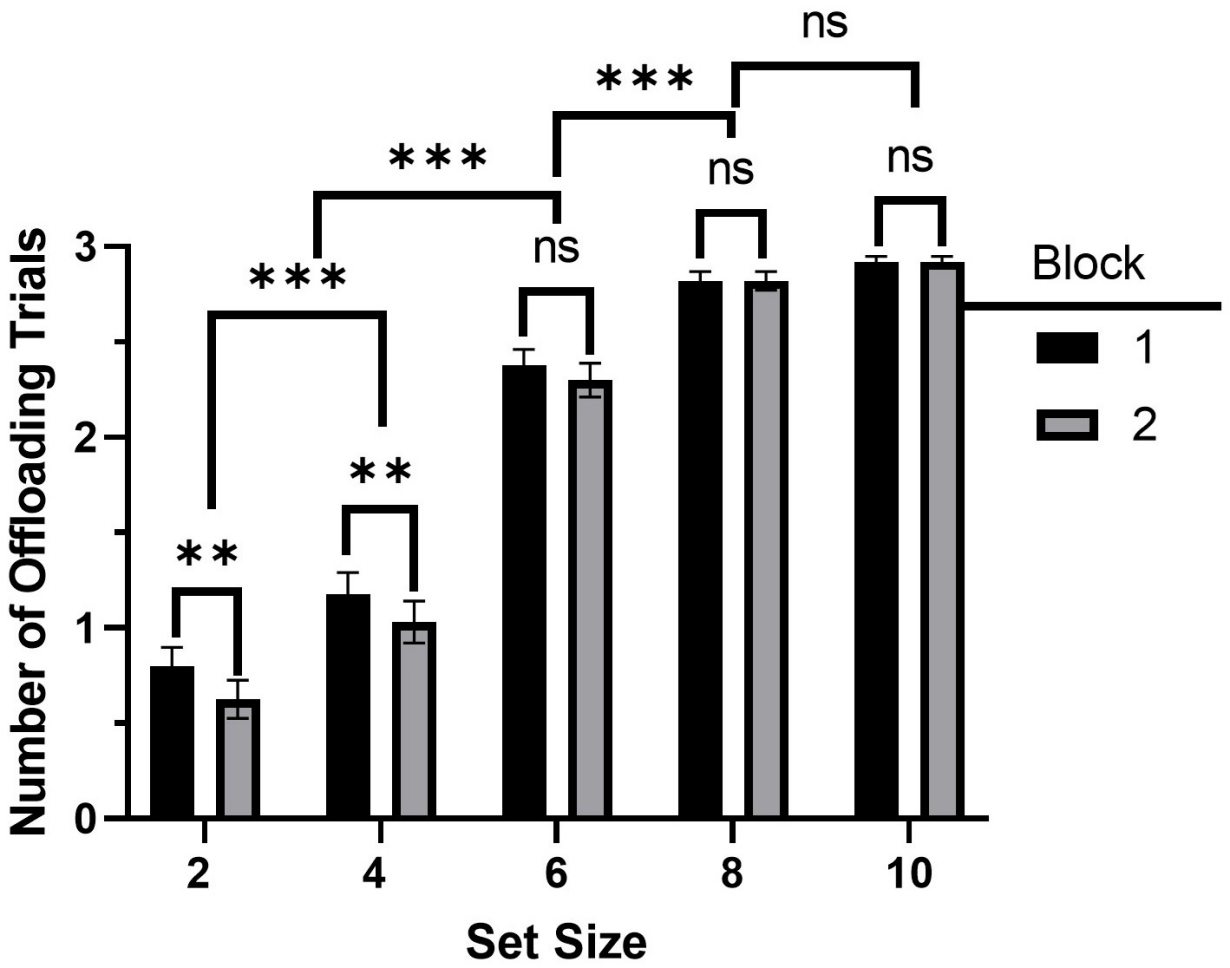
Mean number of trials for which participants wrote down letters prior to reporting them in first block and second block for each set size. Error bars denote standard errors. (**p ≤ 0.01, ***p < 0.001).

There was also a significant interaction between Block and Set Size, *F*(3.36, 524.00) = 3.42, *p* = 0.014, η*p*^2^ = 0.02 (See Figure 2). To further understand the interaction between Block and Set Size, a series of *post hoc* tests was conducted. Specifically, paired *t*-tests with Bonferroni adjustment for multiple comparisons (critical *p*-value of 0.01) revealed that for Set Sizes of 2 and 4, participants wrote down significantly more letters in Block 1 than Block 2, *t*s(157) > 2.61, *p*s ≤ 0.01, Cohen's *d* = 0.29 and 0.21, respectively. For Set Sizes of 6, 8 and 10 the effect was not significant, *t*s(157) < 1.48, *p*s > 0.14, Cohen's *d*s < 0.12. In sum, participants became more selective in what they wrote down as the task progressed, being more likely to keep writing down letters in the most challenging conditions while decreasing it in the least challenging conditions.

**Figure 3:**
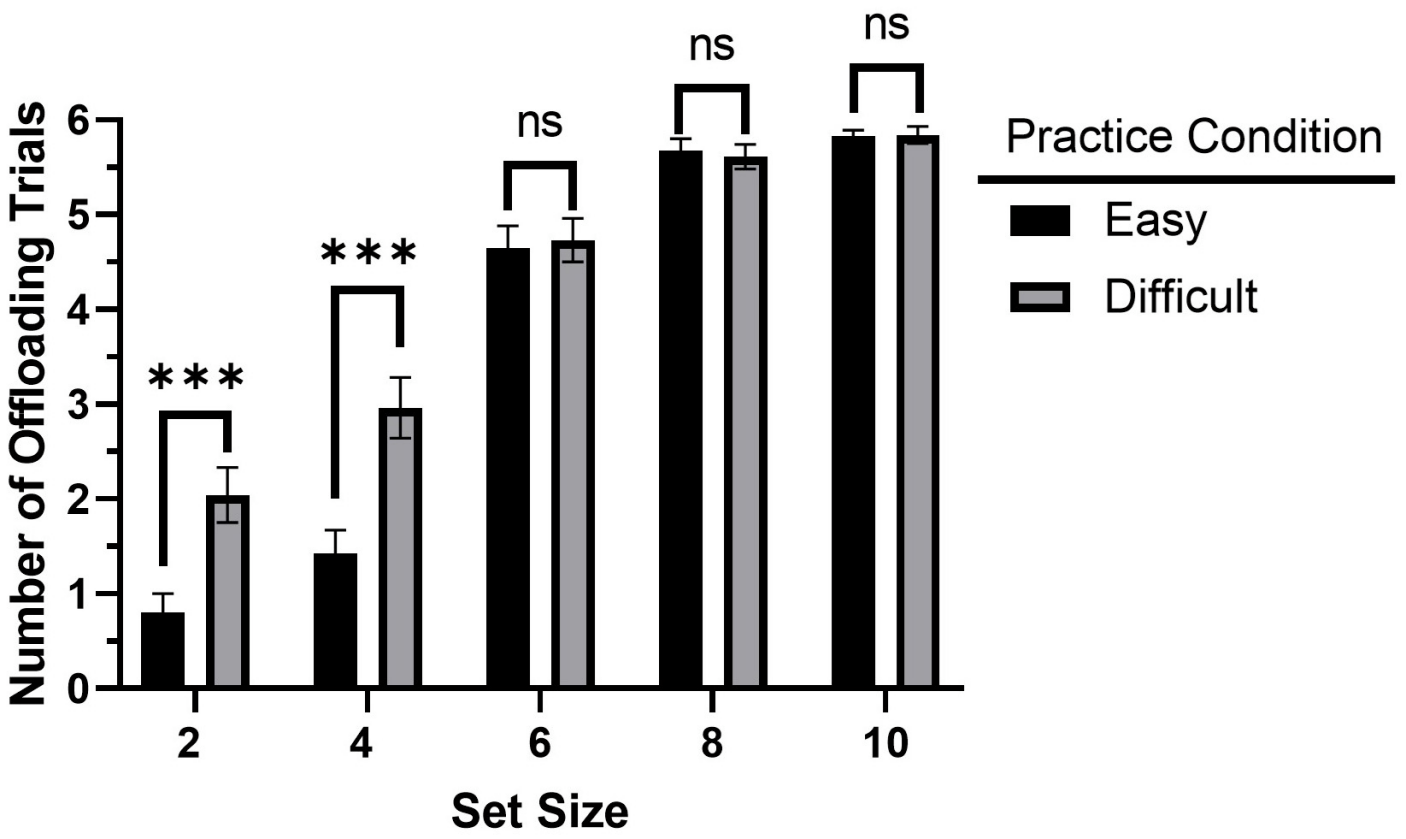
Mean number of trials for which participants in the Easy and Difficult practice condition offloaded letters for each set size. Error bars denote standard errors. (***p < 0.001).

The ANOVA also found an effect of Practice Condition, *F*(1,156) = 7.19, *p* = 0.008, η*p*^2^ = 0.04, with participants offloading more in the experimental trials following the Difficult practice (*M* = 2.12, *SE* = 0.07) than the Easy practice (*M* = 1.84, *SE* = 0.08), supporting H1b. This was modified by a significant interaction between Set Size and Practice Condition, *F*(4,624) = 10.46, *p* < 0.001, η*p*^2^ = 0.06. We conducted *post hoc* independent samples *t*-tests with Bonferroni adjustment for multiple comparisons, yielding a critical *p*-value of 0.01. These revealed that for Set Sizes of 2 and 4, those in the Easy practice offloaded less than those in the Difficult practice (2: *M* = 0.80, *SE* = 0.20 vs. *M* = 2.04, *SE* = 0.29; 4: *M* = 1.42, *SE* = 0.25 vs. *M* = 2.96, *SE* = 0.32), *t*s(137.90) > 3.53, *p*s < 0.001, Cohen's *d* = 0.48 and 0.56, respectively. For Set Sizes 6, 8 and 10, however, those in the Easy practice did not offload less than those in the Difficult practice (6: *M* = 4.64, *SE* = 0.23 vs. *M* = 4.73, *SE* = 0.23; 8: *M* = 5.67, *SE* = 0.13 vs. *M* = 5.61, *SE* = 0.13; 10: *M* = 5.83, *SE* = 0.06 vs. *M* = 5.84, *SE* = 0.09), *t*s < 1, Cohen's *d* = 0.01, 0.04, and 0.05, respectively. Thus, although those receiving the Difficult practice tended to offload more in the experimental trials, this was only the case in the two smallest set sizes (See Figure 3).

Finally, the ANOVA failed to find a significant interaction between Block and Practice Condition, *F* < 1, η*p*^2^ = 0.005 and between Practice Condition, Block, and Set Size, *F* < 1, η*p*^2^ = 0.002. Although, as we saw earlier, participants generally offloaded less in Block 2 particularly for Set Sizes 2 and 4, this is not due to those in the Difficult practice condition offloading less as the task progressed.

### 3.2 Accuracy performance

To examine whether the opportunity to offload the letters and type of practice affected accuracy at reporting them, we carried out a 2 x 2 x 2 x 5 mixed ANOVA with Memory Condition (Choice or No Choice), Block (Block 1 or Block 2) and Set Size (Set Size 2, 4, 6, 8 or 10) as repeated-measures factors, and Practice Condition (Easy or Difficult) as the between-subjects factor. The DV is the proportion of letters accurately reported. Where Mauchly's test of sphericity was significant, the Huynh-Feldt or Greenhouse-Geisser correction was used, as appropriate.

The ANOVA revealed a main effect of Memory Condition, *F*(1,156) = 1,400.00, *p* < 0.001, η*p*^2^= 0.90, consistent with our predictions for H2a. Participants were much more accurate in the Choice (*M* = 0.85, *SE* = 0.01) than in the No Choice condition (*M* = 0.46, *SE* = 0.01), demonstrating the clear benefit of offloading. There was also a main effect of Set Size, *F*(3.34, 521.65) = 1,137.30, *p* < 0.001, η*p*^2^= 0.88 (Set Size 2: *M* = 0.98, *SE* = 0.003; Set Size 4: *M* = 0.92, *SE* = 0.008; Set Size 6: *M* = 0.60, *SE* = 0.01; Set Size 8: *M* = 0.43, *SE* = 0.01; Set Size 10: *M* = 0.34, *SE* = 0.01). Pairwise comparisons with Bonferroni adjustment for multiple comparisons (critical *p*-value = 0.005) revealed that each of these means are significantly different from the others (all *p*s < 0.001). As Set Size increased, accuracy decreased. No other main effects were significant, including the effect of Block, *F* < 1, η*p*^2^= 0.005 and the effect of Practice condition, *F*(1,156) = 1.02, *p* = 0.32, η*p*^2^= 0.006. Thus, although people in the Difficult practice offloaded more often, this did not translate into overall greater accuracy on their part. This is likely because they were more likely to offload compared to the Easy practice only in the easiest set sizes, which had very high accuracies.

**Figure 4:**
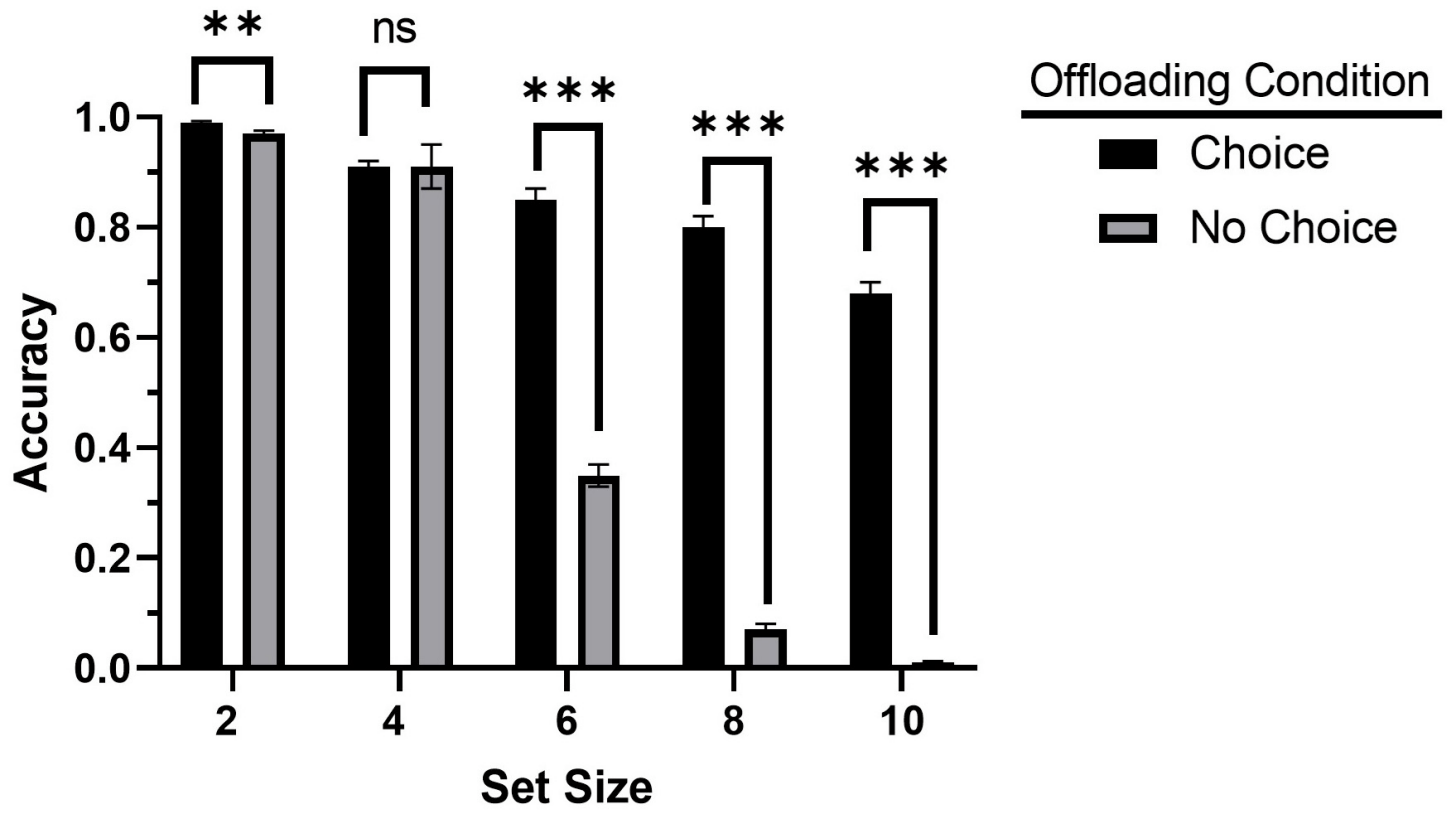
Accuracy in the Choice and No Choice conditions in each set size. Error bars denote standard errors. (**p < 0.01; ***p < 0.001).

The ANOVA also revealed an interaction between Memory Condition and Set Size, *F*(3.36,524.19) = 444.01, *p* < 0.001, η*p*^2^= 0.74, supporting H2b. Paired *t*-tests with Bonferroni adjustment for multiple comparisons (critical *p*-value of 0.01) revealed that for Set Size 2, participants were slightly more accurate in the Choice (*M* = 0.99, *SE* = 0.003) than in the No Choice condition (*M* = 0.97, *SE* = 0.005), *t*(157) = 2.90, *p* = 0.004, Cohen's *d* = 0.23. For Set Size 4, participants did not differ in accuracy in the Choice (*M* = 0.91, *SE* = 0.01) and the No Choice (*M* = 0.92, *SE* = 0.01) conditions, *t* < 1, Cohen's *d* = 0.01. For Set Size 6, accuracy was higher in the Choice (*M* = 0.85, *SE* = 0.02) than in the No Choice condition (*M* = 0.35, *SE* = 0.02), *t*(157) = 21.14, *p* < 0.001, Cohen's *d* = 1.68. In Set Size 8, accuracy was also higher in the Choice (*M* = 0.80, *SE* = 0.02) than the No Choice condition (*M* = 0.07, *SE* = 0.01), *t*(157) = 33.89, *p* < 0.001, Cohen's *d* = 2.70. Finally, in Set Size 10, accuracy was also higher in the Choice (*M* = 0.68, *SE* = 0.02) than the No Choice condition (*M* = 0.01, *SE* = 0.003), *t*(157) = 31.87, *p* < 0.001, Cohen's *d* = 2.54. Evidently, as shown in Figure 4, the largest effect on accuracy of being able to offload the letters was in the higher set sizes.

**Figure 5:**
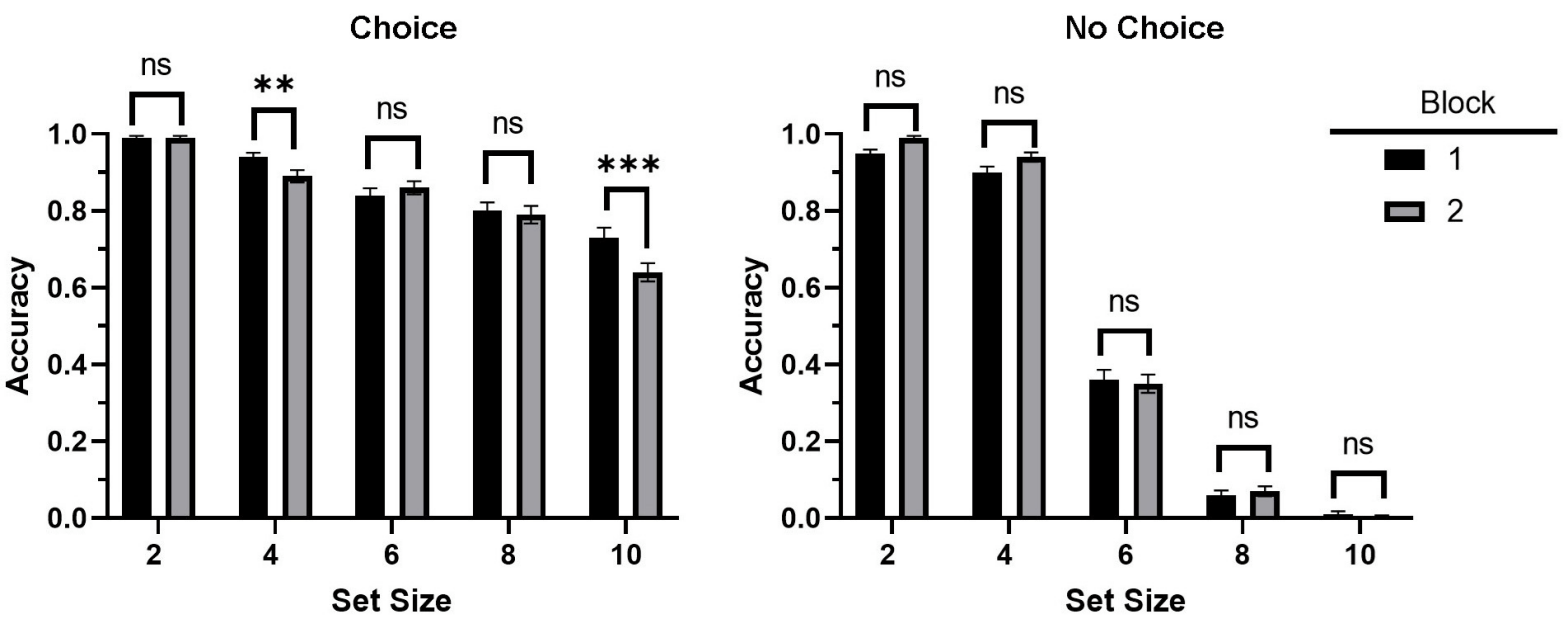
Accuracy for each Block and Set Size in the Choice **(left panel)** and No Choice **(right panel)** condition. Error bars denote standard errors. (**p < 0.01; ***p < 0.001).

The ANOVA also revealed an interaction between Block and Set Size, *F*(3.53, 551.28) = 3.52, *p* = 0.01, η*p*^2^= 0.02 and an interaction between Memory Condition and Block, *F*(1,156) = 12.90, *p* < 0.001, η*p*^2^= 0.08. These interactions were modified by a three-way interaction between Memory Condition, Block, and Set Size, *F*(3.32, 518.33) = 4.13, *p* = 0.005, η*p*^2^= 0.03 (see Figure 5). Breaking this interaction down, in the No Choice condition, the interaction between Block and Set Size was not significant, *F*(2.39, 375.59) = 2.05, *p* = 0.12, η*p*^2^= 0.01. This means that the difference in accuracy from first to second block did not differ across set sizes (See Figure 5 right panel).

In the Choice condition, the interaction between Block and Set Size was significant, *F*(3.30, 518.33) = 4.95, *p* = 0.001, η*p*^2^= 0.03 (See Figure 5 left panel). Paired *t*-tests with Bonferroni adjustment for multiple comparisons (critical *p*-value of 0.01) revealed that for Set Size 2 there was no difference in accuracy between Block 1 (*M* = 0.99, *SE* = 0.01) and Block 2 (*M* = 0.99, *SE* = 0.01), *t* < 1, Cohen's *d* = 0.02. For Set Size 4, Block 1 (*M* = 0.94, *SE* = 0.01) did differ from Block 2 (*M* = 0.89, *SE* = 0.02), *t*(157) = 2.90, *p* = 0.004, Cohen's *d* = 0.23. For Set Size 6, Block 1 (*M* = 0.84, *SE* = 0.02) and Block 2 (*M* = 0.86, *SE* = 0.02) did not differ, *t*(157) = 1.23, *p* = 0.22, Cohen's *d* = 0.10, and for Set Size 8, Block 1 (*M* = 0.80, *SE* = 0.02) also did not differ from Block 2 (*M* = 0.79, *SE* = 0.02), *t* < 1, Cohen's *d* = 0.04. For Set Size 10, accuracy was higher in Block 1 (*M* = 0.73, *SE* = 0.03) than in Block 2 (*M* = 0.64, *SE* = 0.02), *t*(157) = 3.32, *p* = 0.001, *d* = 0.26. Moreover, in the Choice condition there was a significant effect of Set Size, *F*(3.29, 516.11) = 79.50, *p* < 0.001, η*p*^2^= 0.34, and a significant effect of Block, such that participants were more accurate in the first block (*M* = 0.86, *SE* = 0.01) than in the second block (*M* = 0.83, *SE* = 0.01), *F*(1,157) = 8.73, *p* = 0.004, η*p*^2^= 0.05.

In short, only for two set sizes there was a drop in accuracy from first block to the second block in the Choice condition. The drop in accuracy for Set Size 4 could be accounted for by the fact that offloading decreased from the first to the second block in that condition. This was not the case, however, for Set Size 10, where the biggest drop in accuracy was evident, as people offloaded the same amount in both blocks of that condition. In contrast, in the No Choice condition, the difference in accuracy between blocks was consistent across set sizes.

No other main effects or interactions were significant in the 2 x 2 x 2 x 5 mixed ANOVA.

### 3.3 Analyses of individual differences in STM and offloading

As noted, STM was calculated by mean accuracy in the No Choice condition. The mean STM score was 0.46 (*SE* = 0.01), with scores ranging from 0.30 to 0.70. Overall, STM did not correlate with offloading frequency (*r* = −0.15, *p* = 0.061). However, we were agnostic regarding correlations with offloading overall and instead predicted that STM would correlate negatively with offloading frequency only at intermediate set sizes. To assess this, we carried out correlational analyses between STM and offloading frequency at each of the five set sizes, with Bonferroni correction for multiple tests (critical *p*-value = 0.01). The results of these analyses are listed in Table 1.

**Table 1:** Pearson correlations (*r*) between STM and number of times participants wrote down letters prior to reporting them at each set size.

**Set size:**	**SS2**	**SS4**	**SS6**	**SS8**	**SS10**
Correlation	−0.05 (0.57)	−0.07 (0.41)	**−0.20** ^*^ **(0.01)**	−0.18 (0.026)	−0.16 (0.042)

Numbers in parentheses are p-values. (^*^p ≤ 0.01). Significant effects appear in bold text.

These analyses revealed that, as we predicted, STM capacity was only significantly correlated with offloading frequency for the intermediate set size 6, supporting H3. When the set size was 2 or 4, and the task was relatively easy, STM did not predict offloading, likely as few people had to offload. At set sizes 8, and 10, STM also did not significantly correlate with frequency of offloading after correcting for multiple tests. These results are consistent with Risko and Dunn (2015) and inconsistent with Richmond et al. (2025).

### 3.4 Metacognitive judgements and self-ratings

We turn now to the metacognitive judgments and self-ratings of effort and motivation for which questions and descriptive statistics are listed in Table 2. We examined metacognitive judgments and self-ratings to test whether confidence regarding how well one will do on the Choice task, effort to offload the letters after the Choice trials, and motivation to correctly report the letters predict frequency of offloading. To this end, we carried out a simultaneous regression to predict overall offloading frequency using these three variables as predictors. To test for multicollinearity, we checked for correlations between the three predictors to see if any were greater than *r* = 0.80. We found that confidence ratings did not correlate with effort (*r* = −0.10, *p* = 0.246) or with motivation (*r* = +0.13, *p* = 0.127). And, although effort did correlate with motivation (*r* = +0.21, *p* = 0.008), this was well below the threshold for multicollinearity concerns. The overall regression model was significant and predicted 6% of the variance in offloading frequency, *F*(3,145) = 3.33, *p* = 0.02, *R*^2^ = 0.064. However, only motivation was a significant positive predictor of offloading frequency (See Table 3), supporting H4b but not H4a or H4c.^2^ Variance inflation factors suggested that multicollinearity was not a concern (confidence = 1.03, effort = 1.06, motivation = 1.07).

**Table 2:** Self-rating questions asked during Letter-Naming task, each rated on a 1–7 scale.

**Question**	**Time assessed**	**Mean (out of 7 max)**	**Range**	***SE***
Q1. How accurate do you think you will be?	After practice, before Choice experimental trials	5.05	1.00–7.00	0.10
Q2. How often will you write down the letters?	After practice, before Choice experimental trials	5.63	2.00–7.00	0.10
Q3. How much effort will it be to write letters?	After practice, before Choice experimental trials	3.52	1.00–7.00	0.15
Q4. How well do you think you performed?	After Choice experimental trials	5.30	2.00–7.00	0.09
Q5. How good is your short-term memory?	After Choice experimental trials	3.66	1.00–7.00	0.10
Q6. How much effort was it to write letters?	After Choice experimental trials	3.12	1.00–7.00	0.15
Q7. How motivated were you to correctly report the letters?	After Choice experimental trials	5.96	2.00–7.00	0.09
Q8. How well do you think you performed?	After No Choice experimental trials	2.70	1.00–7.00	0.09
Q9. How good is your short-term memory?	After No Choice experimental trials	2.73	1.00–7.00	0.10
Q10. How motivated were you to correctly report the letters?	After No Choice experimental trials	5.13	1.00–7.00	0.13

**Table 3:** Regression table.

**Variable**	**Unstandardized beta**	***SE***	**Standardized beta**	***p***	**95% CI**	**Partial correlation**
Confidence	0.54	0.45	0.10	0.23	−0.35, 1.43	0.10
Offloading effort	0.28	0.30	0.08	0.36	−0.32, 0.87	0.08
Accuracy motivation	1.13	0.47	0.20	**0.02** ^*^	0.20, 2.07	0.20

Confidence = How well will you perform in the upcoming trials? Effort = How much effort will it be to write down the letters as you hear them? Motivation = How motivated were you to correctly report the letters? DV = offloading frequency. ^*^ = *p* < 0.05. Significant effects appear in bold text.

The self-ratings of effort also allowed us to test the hypothesis that the greater offloading following the Difficult practice stems from the perseveration of a preferred strategy developed during practice and not due to offloading becoming easier due to practice. Independent samples *t*-tests revealed that those in the Difficult practice condition did not give higher ratings to how much effort it will be to write down the letters (*M* = 3.71, *SE* = 0.21), compared to those in the Easy practice condition (*M* = 3.33, *SE* = 0.21), *t*(154) = 1.30, *p* = 0.196, Cohen's *d* = 0.21. They did, however, give higher ratings to their likelihood of writing down the letters in the upcoming trials (*M* = 6.24, *SE* = 0.12) compared to those in the Easy practice condition (*M* = 4.99, *SE* = 0.14), *t*(153) = 6.90, *p* < 0.001, Cohen's *d* = 1.11. This pattern of results suggests that the best explanation for the effects of Practice Condition on offloading is due to perseveration, and not due to a practice effect where offloading becomes easier due to practice. Thus, H4d was supported. The perseveration is uniquely due to the Difficult practice, as 24% of those participants went on to offload on every trial of the Choice condition, while only 6% of those in the Easy practice did so. Moreover, none of those in the Easy practice refused to offload in the experimental trials, and only 1% of those in the Difficult practice condition did so.

The metacognitive judgments and self-ratings of effort and motivation also allowed us to compare the Choice and No Choice conditions to see how the opportunity to offload influences participants' estimates of their memory abilities, motivation to respond correctly, and assessment of their performance. Paired sample *t*-tests with Bonferroni adjustment for multiple comparisons (critical *p*-value = 0.0167) revealed that, not surprisingly, participants' assessment of how well they did was higher (*M* = 5.30, *SE* = 0.09) following the Choice trials than following the No Choice trials (*M* = 2.70, *SE* = 0.09), *t*(157) = 22.67, *p* < 0.001, Cohen's *d* = 1.81. Given that accuracy was higher in the Choice condition, this reflects an awareness of how offloading can facilitate memory performance. Furthermore, following the Choice condition, participants reported being more motivated to correctly report the letters (*M* = 5.96, *SE* = 0.09) than following the No Choice trials (*M* = 5.13, *SE* = 0.13), *t*(157) = 7.76, *p* < 0.001, Cohen's *d* = 0.62, supporting H4f. Finally, although the self-ratings of short-term memory following the Choice condition were correlated with those following the No Choice condition (*r* = +0.58, *p* < 0.001), following the Choice condition, participants gave higher ratings to their short-term memory ability (*M* = 3.66, *SE* = 0.10) than following the No Choice condition (*M* = 2.74, *SE* = 0.10), *t*(156) = 10.09, *p* < 0.001, Cohen's *d* = 0.81, supporting H4e and consistent with the claim that the ability to offload can inflate the assessment of one's cognitive abilities. Interestingly, ratings of short-term memory following Choice condition were unrelated to performance in the Choice condition (*r* = + 0.07, *p* = 0.40), though self-ratings of short-term memory following No Choice condition were correlated with performance therein (*r* = +0.28, *p* < 0.001).

## 4 Discussion

The present study examined how STM, task characteristics, type of practice, metacognitive judgments of confidence in how well they will do on the task, and self-ratings of effort and motivation affect offloading frequency. These goals were pursued using Risko and Dunn's (2015) Letter-Naming task, a task also used by Morrison and Richmond (2020) and Richmond et al. (2025) to study cognitive offloading.

### 4.1 Offloading as a function of task-characteristics and type of practice

Consistent with our predictions (H1a) and prior findings by Risko and Dunn (2015) and Morrison and Richmond (2020), we found that as set size increased, so did the frequency with which people offloaded the letters prior to reporting them. This can be explained by Gilbert's (2024) decision-making model because it predicts that when set sizes are small, people should be less willing to pay the costs associated with offloading, but as the task becomes more challenging people should be more willing to pay the offloading cost.

With respect to the effects of type of practice, which has not been studied in the context of the Letter-Naming task, we found that those receiving the Difficult practice offloaded more than did those receiving the Easy practice, consistent with our predictions (H1b). The effects of Practice Condition were particularly evident in the smallest two set sizes, as Practice Condition interacted with Set Size. Thus, although Difficult practice yielded more offloading, this was particularly evident in the smallest set sizes. Our results are consistent with prior studies looking at the effect of practice difficulty on offloading behavior, particularly those by Patrick et al. (2015) using the Blocks World Task and Weis and Kunde (2024) using a stimulus rotation task. Both studies found that participants who were given the option to either rely on internal or external (i.e., offloading) strategies to carry out a task were more likely to continue relying on external strategies in transfer trials when first trained using a difficult practice condition that encourages offloading. Weis and Kunde (2024) found this to be the case even though in the transfer trials both the internal and external strategies were designed to be equally effective. They describe this as strategy perseveration, where people develop a preference during training and then continue to use the preferred strategy to avoid incurring further decision-making and task-switching costs. They found this to be the case particularly with those exposed to difficult practice.

Our results also demonstrate a perseveration effect unique to those in the Difficult practice condition. Indeed, almost a quarter of those in the Difficult practice continued to offload for all the experimental trials in both blocks. A similar perseveration of response strategy was not evident for those in the Easy practice, a finding consistent with that of Weis and Kunde (2024). Those in the Easy practice were more likely to subsequently tailor their response strategy to the demands of the task—i.e., they were less likely to offload during the smallest two set sizes, which are easy to respond to using internal memory alone. Moreover, although those in the Difficult practice condition offloaded more often during the practice trials, this added practice did not make it easier to subsequently offload the letters, which would suggest a different interpretation of our findings. This is evidenced by the fact that both practice groups gave equal ratings to how difficult it will be to offload the letters after the practice trials. Nonetheless, consistent with H4d, those in the Difficult practice gave higher ratings to the likelihood that they would offload the letters in the upcoming trials than did those in the Easy practice.

An important implication of our study is that if a training program seeks to prevent operators developing a rigid response strategy to limit decision-making and task-switching costs, it is important to have an appropriate training regimen. Such a regimen would be one that exposes individuals to the full range of conditions that they will encounter. This is more likely to encourage a flexible response strategy, one that is more sensitive to specific task conditions, and the costs and benefits associated with internal and external cognitive strategies.

With respect to accuracy on the Letter-Naming task, as we predicted (H2a), we found that accuracy was much higher in the Choice condition than in the No Choice condition. Moreover, accuracy decreased with increasing set size. This effect, however, was much greater in the No Choice condition, supporting H2b. The opportunity to offload therefore kept accuracy on the task relatively high, including in the more difficult conditions. This is consistent with many prior studies demonstrating the consequences of cognitive offloading (e.g., Dupont et al., 2023; Morrison and Richmond, 2020; Richmond et al., 2025; Risko and Dunn, 2015; Storm and Stone, 2015). Although we found that people offloaded more in the first block of trials than in the second, this was not reflected in differences in accuracy between the two blocks of the Letter-Naming task. Block did, however, interact with Set Size and Memory Condition. Differences in accuracy between blocks at each set size were more evident in the Choice condition (i.e., Set Sizes 4 and 10) than in the No Choice condition (i.e., no effect of Block for any set size). Failure to find a difference between two blocks for Set Size 10 of the No Choice condition is likely due to floor effects, as accuracy was very low.

### 4.2 STM capacity and offloading

The central aim of our study was to adjudicate between the conflicting results of Risko and Dunn (2015), Morrison and Richmond (2020) and Richmond et al. (2025) with respect to whether internal memory capacity correlates with frequency of offloading. As noted, Risko and Dunn (2015) found that STM capacity, assessed by average performance in the No Choice task, was correlated with the overall frequency of participants offloading during the Letter-Naming task. Morrison and Richmond (2020), using a much larger sample size, failed to find that STM capacity predicted overall frequency of offloading, and neither did their measures of WM capacity. These papers, however, failed to examine relations between STM and WM and offloading at specific set sizes. Richmond et al. (2025) did attempt to address this issue using the Letter-Naming task, but their Letter-Naming task omitted the highest set size, making it easier for participants, and did not examine relations at each set size, instead grouping them into High and Low memory load conditions. Furthermore, they did not measure STM specifically, but rather WM. Although STM is held to be a component of WM, tasks that measure WM include additional processing requirements that prevent us from specifically targeting the storage capacity of the memory buffers using such measures.

Instead, we used the original Letter-Naming task and tested correlations between STM and offloading at each of the five set sizes, not just with offloading overall. Although we were agnostic about whether there would be a negative correlation between STM capacity and frequency of offloading overall, we did predict that the most robust correlations would be evident only for the intermediate set sizes. When the task is too easy, very few people would offload because the task can readily be done with internal memory, making a relationship between STM and offloading unlikely. When the task is very difficult and memory demands exceed the capacity of most individuals, a relationship between STM and offloading frequency was also not expected.

We found that the correlation between STM capacity and overall frequency of offloading was not significant. In terms of specific set sizes, we found significant negative correlations only for Set Size 6, after correcting for multiple tests, consistent with our predictions (H3). STM capacity did not correlate with frequency of offloading for the two smallest set sizes or for the largest two set sizes. When examining the relevance of STM capacity for cognitive offloading, it is therefore of crucial importance to consider specific task demands, as these can either illuminate or obscure the importance of this cognitive mechanism. Clearly, a rough metric of collapsing set sizes into two groups as was done by Morrison and Richmond (2020) is not sufficiently fine-grained.

Our findings regarding STM and offloading therefore support Gilbert's (2024) value-based decision-making model. According to this model, the more limited a person's internal memory capacity, the greater the opportunity-costs associated with storing information internally. This yields a greater payoff for offloading information, but only when the task demands are sufficiently challenging.

### 4.3 Metacognitive judgments and self-ratings

Participants' ratings of how well they will perform in the upcoming experimental trials were used as an estimate of their metacognitive confidence. We examined whether it predicts overall frequency of offloading after controlling for effort and motivation. Prior studies examining prospective memory on an intention offloading task found that participants who are less confident are more likely to offload intentions (e.g., Boldt and Gilbert, 2019; Scott and Gilbert, 2024; Gilbert et al., 2020; and Hu et al., 2019). Using the Letter-Naming task, however, Richmond et al. (2025) failed to find that confidence predicted offloading frequency. Using a more difficult version of the task, we also failed to find that confidence ratings predict overall frequency of offloading (i.e., no support for H4a). However, unlike prior studies, we controlled for motivation to respond correctly and effort to offload the letters.

Moreover, in the same analysis we failed to find that participants' ratings of how much effort it was to write down the letters as they heard them predicted frequency of offloading (i.e., not support for H4c). Gilbert's decision-making model predicts that this factor should play an important role in the decision to offload, and it is unclear why we failed to find support in terms of this prediction, despite other results supporting the model. Although it could be argued that writing down letters as one hears them is not very difficult, we note that the mean rating of effort was 3.12 out of 7, suggesting that participants did find offloading at least somewhat effortful. We did, however, find that participants' motivation to accurately report the letters accounted for a significant amount of the variance in offloading frequency, supporting H4b. The higher the participants' self-ratings of motivation to correctly report the letters, the more often they offloaded overall. This result, not previously reported in the offloading literature, should be considered in future studies looking at factors influencing strategy choice.

Our metacognitive judgments and self-ratings also produced results regarding people's estimation of their own memory abilities and performance on the task. Not surprisingly, we found that participants gave higher ratings to their performance following the Choice condition than following the No Choice condition, even though they were not given explicit feedback regarding their accuracy. This shows that people are generally aware of the benefits of offloading information, as found by Risko and Dunn (2015), and that the absence of external memory supports can undermine performance.

Moreover, we found that participants' ratings of their short-term memory ability were lower following the No Choice than the Choice condition (supporting H4e) and that people were less motivated to report the letters correctly following the former condition, supporting H4f.

One possible explanation of the higher ratings following the Choice condition stems from the extended mind hypothesis, according to which people form extended cognitive systems with external tools and other people (e.g., Clark, 2008; Sparrow et al., 2011). As “natural born cyborgs,” people are adept at incorporating external devices as they engage in complex cognitive tasks (Clark, 2004). There is evidence that this reliance on external resources can degrade internal cognitive functions (see Wilmer et al., 2017 for a review of research on how reliance on external tools can undermine memory) and thereby result in a lower assessment of one's cognitive abilities when these resources are absent (e.g., Ward, 2013). These claims should be interpreted with caution, however, because although we explicitly asked people to rate their short-term memory ability, it is likely that people understand this term differently than cognitive scientists do. In addition, because participants were asked to rate their memory after each condition, we do not know if their judgments are based on all the trials in the conditions or only some of them. Thus, higher ratings following the Choice condition could be based on an accurate assessment for those trials in which they chose not to offload.

### 4.4 Limitations and future directions

The present study focused specifically on STM and its relation to offloading in a Letter-Naming task. This was done to isolate the internal storage system, limitations of which are expected to play a crucial role in the decision to offload information according to Gilbert's (2024) model. Most prior studies have made use of WM tasks, but WM includes executive functions in addition to the STM stores. Nonetheless, future studies should include several measures of both STM and WM to isolate both components and examine their role in cognitive offloading. It is possible, for example, that variability in the executive functions can account for whether people develop a perseveration strategy following the Difficult practice condition. Indeed, research has shown that individuals who perform worse on measures of executive control are more likely to perseverate (e.g., Dehais et al., 2019).

Moreover, future studies examining how removing the opportunity to offload influences assessments of one's mental abilities in the Letter-Naming task should also use a broader metric such as the Cognitive Self-Esteem (CSE) test (Ward, 2013). We relied on a single question asking about the strength of their short-term memory. Cognitive Self-Esteem is a complex construct that includes assessments of one's thinking ability, internal memory capacity, and ability to access external information when needed. In addition, this should be assessed prior to the Letter-Naming task to get an adequate baseline. In the current study we only assessed self-ratings of memory ability following the two memory conditions.

Further studies should also carry out more fine-grained assessments of metacognitive confidence. Specifically, to properly assess confidence it would be fruitful to collect ratings of confidence in being able to remember items at specific set sizes. Overall judgments of the ability to do well on a task, or overall judgments of one's memory ability may not be robust enough to capture effects of this metacognitive judgment and could account for some of the inconsistent results in the literature. Likewise, judgments of effort involved in offloading should examine how much effort it is to offload letters at specific set sizes instead of gathering an overall rating of effort. Having such ratings would allow for a much more accurate test of Gilbert's (2024) value-based decision-making model.

### 4.5 Conclusion

To conclude, our results support Risko and Dunn (2015) in finding that STM capacity predicts frequency of offloading, at least in intermediate set sizes. Thus, it is important to consider not just overall frequency of offloading as this can obscure the real relation between STM capacity and likelihood of offloading. Consistent with prior studies, we found that the opportunity to offload increases accuracy, especially in the most challenging conditions. Our experiment also showed that the amount of information to be retained influences offloading behavior. In addition, we found that a difficult practice condition can encourage offloading behavior, with many participants developing a perseveration response, offloading the letters more often regardless of task characteristics. Participants' metacognitive judgments and self-ratings also show that the motivation to correctly report the letters on the primary task predicts variability in offloading frequency after controlling for both metacognitive confidence and reported effort of offloading the letters. The latter two factors did not predict offloading in our study, though more research is needed to fully understand how these influence offloading behavior.

## Data Availability

The datasets presented in this study can be found in online repositories. The names of the repository/repositories and accession number(s) can be found below: https://osf.io/9cp52/?view_only=e286647ad0224250a4952ffbdb943393.

## Footnotes

^1^The assessment of self-control, current mood, Big-5 personality, and emotion regulation were included to begin exploring a wider range of individual difference factors that may be relevant to cognitive offloading behavior, a topic that has been ignored. Our exploratory analyses revealed that with very few exceptions, these metrics did not correlate with frequency of offloading. Although the results are not reported here, the data have been posted in the Open Science Framework and can be accessed with the following link: https://osf.io/9cp52/?view_only=e286647ad0224250a4952ffbdb943393.

^2^Readers may be interested in how these variables predict offloading at each set size, not just overall. However, because our hypotheses regarding these metacognitive judgments and self-ratings only pertained to overall offloading frequency, these analyses are not included in this paper. The results of the analyses have been posted in the Open Science Framework and can also be accessed with the following link: https://osf.io/9cp52/?view_only=e286647ad0224250a4952ffbdb943393.

## References

[B1] Baddeley A. D. (2000). “Short-term and working memory,” in The Oxford handbook of memory eds. E. Tulving and F. I. M. Craik, Oxford: Oxford University Press*, (*pp. 77–92). 10.1093/oso/9780195122657.003.0005

[B2] Boldt A. Gilbert S. J. (2019). Confidence guides spontaneous cognitive offloading. Cogn. Res. 4, 2–16. 10.1186/s41235-019-0195-y 31792746 PMC6889107

[B3] Chiappe D. Morgan C. Kraut J. Ziccardi J. Sturre L. Strybel T. Z.. (2016). Evaluating probe techniques and a situated theory of situation awareness. J. Exp. Psychol. Appl. 4, 436–454. 10.1037/xap0000097 27936855

[B4] Clark A. (2004). Natural born cyborgs: Minds, technologies, and the future of human intelligence. Oxford: Oxford University Press.

[B5] Clark A. (2008). Supersizing the Mind: Embodiment, Action, and Cognitive Extension. New York: Oxford University Press. 10.1093/acprof:oso/9780195333213.001.0001

[B6] Cowan N. (2008). What are the differences between long-term, short-term, and working memory? Progress Brain Res. 169, 323–338. 10.1016/S0079-6123(07)00020-9 18394484 PMC2657600

[B7] Dehais F. Hodgetts H. M. Causse M. Behrend J. Durantin G. Tremblay S. (2019). Momentary lapse of control: a cognitive continuum approach to understanding and mitigating perseveration in human error. Neurosci. Biobehav. Rev. 100, 252–262. 10.1016/j.neubiorev.2019.03.006 30878500

[B8] Dupont D. Zhu Q. Gilbert S. J. (2023). Value-based routing of delayed intentions into brain-based versus external memory stores. J. Exp. Psychol. Gen. 152, 175–187. 10.1037/xge0001261 35913880

[B9] Fellers C. Miyatsu T. Storm B. C. (2023). Remembering what to do when the time comes: the effects of offloading in a complex prospective memory task. J. Exp. Psychol. Appl. 29, 631–644. 10.1037/xap0000449 36201839

[B10] Gilbert S. J. (2015). Strategic use of reminders: influence of both domain-general and task-specific metacognitive confidence, independent of objective memory ability. Conscious. Cogn. 33, 245–260. 10.1016/j.concog.2015.01.006 25666463

[B11] Gilbert S. J. (2024). Cognitive offloading is value-based decision making: modelling cognitive effort and the expected value of memory. Cognition 247:105783, 10.1016/j.cognition.2024.105783 38583321

[B12] Gilbert S. J. Bird A. Carpenter J. Fleming S. M. Sachdeva C. Tsai P.-C. (2020). Optimal use of reminders: metacognition, effort, and cognitive offloading. J. Exp. Psychol. Gen. 149, 501–517. 10.1037/xge0000652 31448938

[B13] Gilbert S. J. Boldt A. Sachdeva C. Scarampi C. Tsai P.-C. (2023). Outsourcing memory to external tools: a review of “intention offloading.” Psych. Bull. Rev. 30, 60–76. 10.3758/s13423-022-02139-4 35789477 PMC9971128

[B14] Grinschgl S. Papenmeier F. Meyerhoff H. S. (2021). Consequences of cognitive offloading: Boosting performance but diminishing memory. Quart. J. Exp. Psychol. 74, 1477–1496. 10.1177/17470218211008060 33752519 PMC8358584

[B15] Hu X. Luo L. Fleming S. M. (2019). A role for metamemory in cognitive offloading. Cognition 193:104012. 10.1016/j.cognition.2019.104012 31271925 PMC6838677

[B16] Kahn A. S. Martinez T. M. (2020). Text and you might miss it? Snap and you might remember? Exploring “Google effects on memory” and cognitive self-esteem in the context of snapchat and text messaging. Comput. Human Behav. 104, 106–166. 10.1016/j.chb.2019.106166

[B17] McCabe D. P. Roediger H. L. McDaniel M. A. Balota D. A. Hambrick D. Z. (2010). The relationship between working memory capacity and executive functioning: evidence for a common executive attention construct. Neuropsychology 24, 222–243. 10.1037/a0017619 20230116 PMC2852635

[B18] Meyerhoff H. S. Grinschgl S. Papenmeier F. Gilbert S. J. (2021). Individual differences in cognitive offloading: a comparison of intention offloading, pattern copy, and short-term memory capacity. Cogn. Res. Princ. Impl. 6:34. 10.1186/s41235-021-00298-x 33928480 PMC8084258

[B19] Morrison A. B. Richmond L. L. (2020). Offloading items from memory: individual differences in cognitive offloading in a short-term memory task. Cogn. Res. Princ. Implic. 5:1. 10.1186/s41235-019-0201-4 31900685 PMC6942100

[B20] Patrick J. Morgan P. L. Smy V. Tiley L. Seeby H. Patrick T.. (2015). The influence of training and experience on memory strategy. Mem. Cognit. 43, 775–787. 10.3758/s13421-014-0501-3 25591501

[B21] Richmond L. L. Burnett L. K. Kearley J. Gilbert S. J. Morrison A. B. Ball B. H. (2025). Individual differences in prospective and retrospective memory offloading. J. Mem. Lang. 142:104617. 10.1016/j.jml.2025.104617 40667475 PMC12263121

[B22] Risko E. F. Dunn T. L. (2015). Storing information in-the-world: metacognition and cognitive offloading in a short-term memory task. Conscious. Cogn. 36, 61–74. 10.1016/j.concog.2015.05.014 26092219

[B23] Risko E. F. Gilbert S. J. (2016). Cognitive offloading. Trends Cogn. Sci. 20, 676–688. 10.1016/j.tics.2016.07.002 27542527

[B24] Schönbrodt F. D. Perugini M. (2013). At what sample size do correlations stabilize? J. Res. Pers. 46, 609–612. 10.1016/j.jrp.2013.05.009

[B25] Scott A. E. Gilbert S. J. (2024). Metacognition guides intention offloading and fulfillment of real-world plans. J. Exp. Psychol. Appl. 30, 539–553. 10.1037/xap0000515 39023988

[B26] Sparrow B. Liu J. Wegner D. M. (2011). Google effects on memory: cognitive consequences of having information at our fingertips. Science 333, 476–478. 10.1126/science.1207745 21764755

[B27] Storm B. Stone S. (2015). Saving-enhanced memory: the benefits of saving on the learning and remembering of new information. Psychol. Sci. 26, 182–188. 10.1177/0956797614559285 25491269

[B28] Ward A. F. (2013). Supernormal: how the internet is changing our memories and our minds. Psychol. Inq. 24, 341–348. 10.1080/1047840X.2013.850148

[B29] Weis P. P. Kunde W. (2024). Switching between different cognitive strategies induces switch costs as evidenced by switches between manual and mental object rotation. Sci. Rep. 14:6217. 10.1038/s41598-024-56836 38485965 PMC10940645

[B30] Weis P. P. Wiese E. (2019). Problem solvers adjust cognitive offloading based on performance goals. Cogn. Sci. 43*:*e12802. 10.1111/cogs.12802 31858630

[B31] Wilmer H. H. Sherman L. E. Chein J. M. (2017). Smartphones and cognition: a review of research exploring the links between mobile technology habits and cognitive functioning. Front. Psychol. 25:8:605. 10.3389/fpsyg.2017.00605 28487665 PMC5403814

